# PTTG: an important target gene for ovarian cancer therapy

**DOI:** 10.1186/1757-2215-1-6

**Published:** 2008-10-20

**Authors:** Siva Kumar Panguluri, Casey Yeakel, Sham S Kakar

**Affiliations:** 1 Department of Physiology and Biophysics, James Graham Brown Cancer Center, University of Louisville, Louisville, KY 40202, USA

## Abstract

Pituitary tumor transforming gene (PTTG), also known as securin is an important gene involved in many biological functions including inhibition of sister chromatid separation, DNA repair, organ development, and expression and secretion of angiogenic and metastatic factors. Proliferating cancer cells and most tumors express high levels of PTTG. Overexpression of PTTG in vitro induces cellular transformation and development of tumors in nude mice. The PTTG expression levels have been correlated with tumor progression, invasion, and metastasis. Recent studies show that down regulation of PTTG in tumor cell lines and tumors in vivo results in suppression of tumor growth, suggesting its important role in tumorigenesis. In this review, we focus on PTTG structure, sub-cellular distribution, cellular functions, and role in tumor progression with suggestions on possible exploration of this gene for cancer therapy.

## Introduction

Although death from ovarian cancer (OCA) ranks fifth in prevalence, it is the most deadliest among gynecological malignancies. Early diagnosis is essential for preventing OCA fatalities. Treatment options for OCA typically include surgery and chemotherapy. The goal of surgery is to remove most of the cancerous growth. However, depending on the stage of the cancer, some cancer cells may remain following surgery. To eliminate these remaining cells, various adjuvant chemotherapy strategies are employed based on cancer stage, tumor grade, and other health concerns. While effective, chemotherapy treatments are accompanied by undesirable side effects rising from the targeting of rapidly dividing cells, which is a hallmark trait of cancer cells. In this process, healthy cells that also rapidly divide such as blood cells and cells lining the mouth and GI tract are also damaged. To reduce such side effects and increase cellular specificity, a targeted cancer therapy for OCA is necessary that pinpoints etiological characteristics other than high cellular metabolic rate. The major drawback in understanding the etiology of OCA is the availability of an appropriate OCA model. Many laboratories have initiated the development of OCA transgenic mice models. However, to date, there is no report of having an efficient transgenic mouse model to study the mechanism of ovarian tumorigenesis [1-6].

Pituitary tumor transforming gene (PTTG) is an oncogene involved in cell cycle regulation and sister chromatid separation. PTTG is highly expressed in various tumors including ovarian, suggesting that PTTG may function in ovarian tumorigenesis. Initially, PTTG was cloned from rat pituitary tumor and shown to induce cellular transformation *in vitro *and tumor development in nude mice [7]. The expression level of this gene was also found in germ, Leyding, and sertoil cells in testis [8]. Subsequently, the human homologue of PTTG was identified and shown to be overexpressed in Jurkat T cells and leukocytes from patients with myelodysplastic syndromes [9]. Zou et al. [10] identified PTTG as the human securin, which is an important protein for the inactivation of separases and thereby keeps the sister chromatids intact until the onset of anaphase. Extensive research on this gene was performed by many investigators in relation to its overexpression in several endocrine-related tumors including pituitary, thyroid, breast, ovarian, and uterine as well as non-endocrine-related cancers such as pulmonary, gastrointestinal, and those related to the central nervous system [11-19]. The availability of the molecular and functional mechanisms of PTTG and its important role in tumorigenesis in various cancers including OCA is of great interest.

### Structure and distribution

#### A. Gene structure and its homologues

Melmed and his colleagues originally isolated PTTG from rat pituitary tumors ([7]. The rat PTTG gene is composed of five exons and four, introns [8]. Zhang et al. [11] characterized the human homolog using rat PTTG cDNA as a screening probe from a human fetal cDNA library. It was shown to have 85% homology with the coding region of rat PTTG. During the same time, we and two other groups independently cloned and characterized human PTTG [[9,12], and [20]]. Reported sequences from all the groups were identical (GenBank accession numbers AJ223953, AF075242, NM_004219, BC101834, AF095287, and CR457135) except from Lee et al. [20], which was found to be 95% identical (AF062649). The human PTTG gene was found to be localized on chromosome 5 [5q35.1] [21]. Mapping of the human PTTG gene revealed that it contains five exons and four introns, which showed significant similarity to the rat PTTG gene [[8] and [22]].

**Figure 1:**
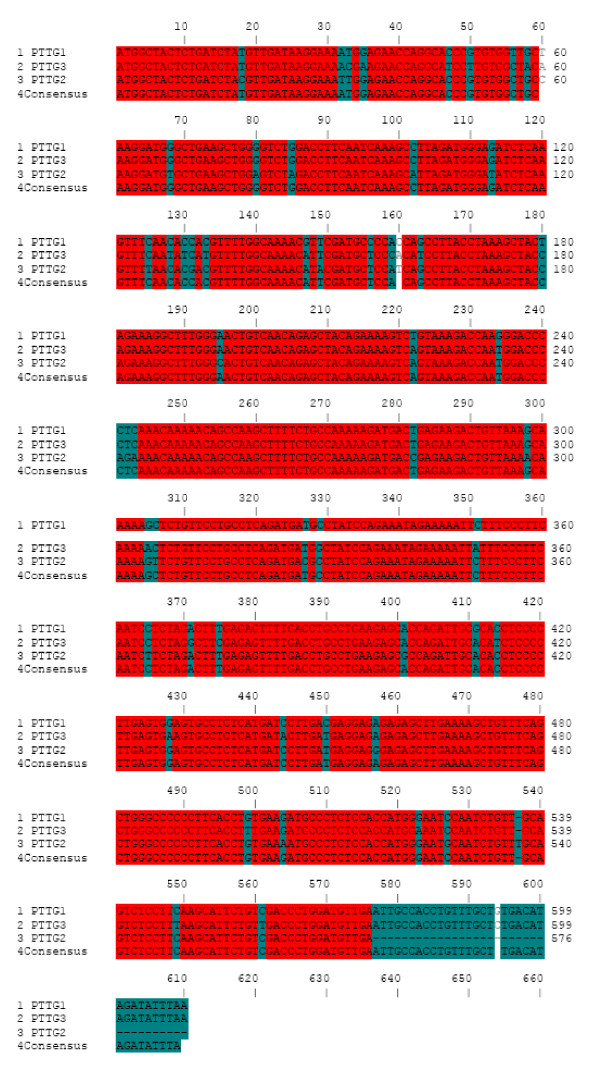
**The cluster analysis of three human PTTG homolog cDNA sequences**. The sequences were analyzed by ANTHEPROT 2000 V6.0. PTTG1, PTTG2 and PTTG3 are the human PTTG isoforms 1, 2 and 3 respectively. The conserved sequences across all these isoforms are shown in row 4. The nucleotide identity is shown in different colors. Red indicates 100% identity, Blue ≥ 75, dark green ≥ 50 and light green < 50.

**Figure 2:**
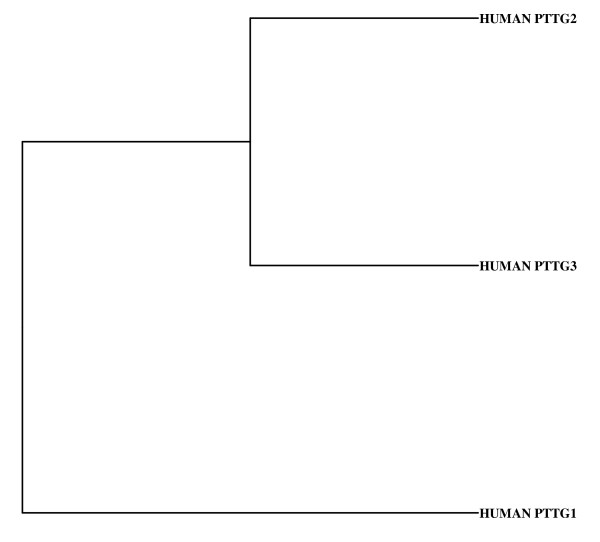
**The Neighbor phylogenetic tree analysis of human PTTG homologues**. PTTG1, PTTG2 and PTTG3 are the human PTTG isoforms 1, 2 and 3 respectively.

Northern blot analysis of PTTG messenger (m)RNA revealed that PTTG mRNA is 1.3 kb with an open reading frame of 609 nucleotides encoding a protein of 203 amino acids (23 kDa). PTTG is a multidomain protein consisting of a transactivation domain, a domain required for ubiquitin-mediated proteolysis, and a DNA-binding domain [23]. Southern blot analysis of human genomic DNA revealed the presence of two additional genes homologous to human PTTG in the genome [24]. The sequencing and restriction map analysis of the additional genes showed significant homology with the PTTG gene. Based on the similarity in the sequences, we renamed PTTG as PTTG1 and the new genes as PTTG2 and PTTG3, respectively. PTTG1 is 91% identical with PTTG2 and 89% identical with PTTG3 at amino acid levels. PTTG2 expression was detected in liver tumors and normal liver tissues. Both the genes were found to be intronless and present on different chromosomes. The PTTG2 gene was localized on chromosome 8 (8q13.1), whereas the PTTG3 gene was present on chromosome 4 (4p15.1) [24]. The cluster analysis of these three PTTG homolog cDNA sequences is shown in Figure 1. The Neighbor phylogenetic tree analyses of human PTTG homologues revealed that PTTG2 and PTTG3 are more similar and formed a cluster leaving PTTG1 separate (Fig. 2). The cDNA sequence of these three homologues showed 85.25% identity to each other. The PTTG2 and PTTG3 cDNA showed 86.56% identity, whereas PTTG1 showed 88.85% identity with PTTG2 and 94.56% identity with PTTG3.

**Figure 3:**
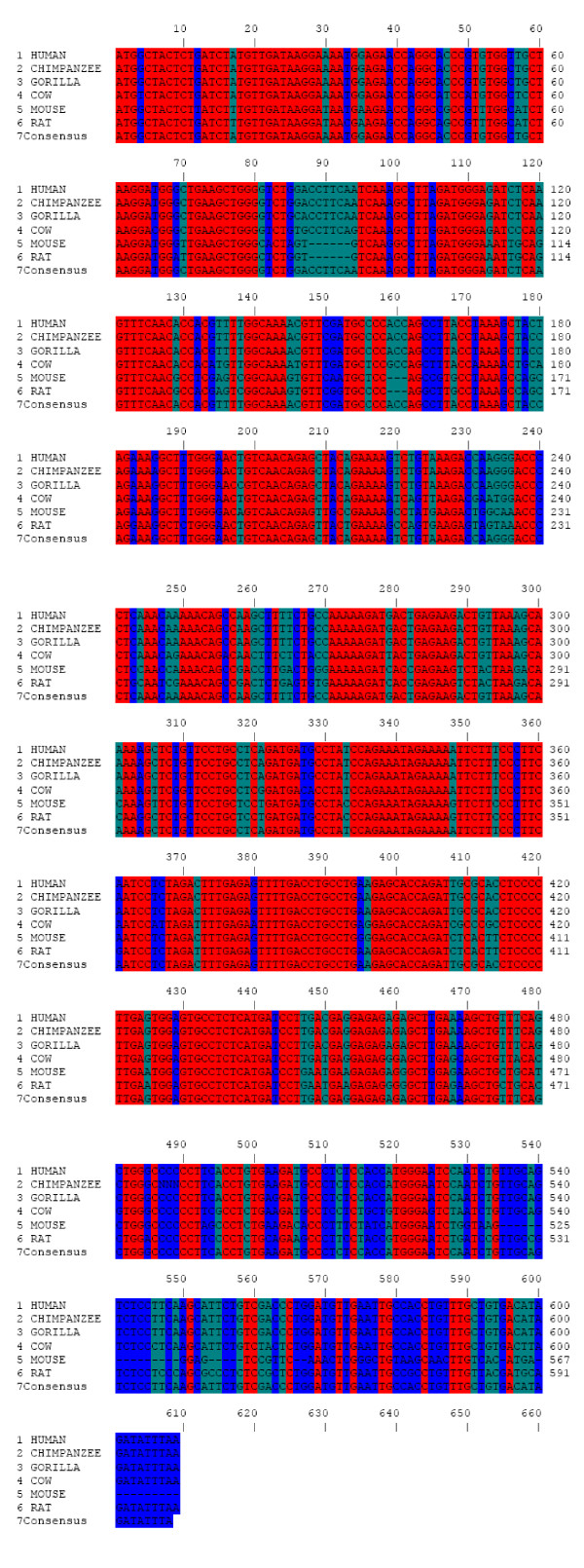
**The cluster analysis of PTTG cDNA sequences from different species**. The sequences were analyzed by ANTHEPROT 2000 V6.0. PTTG cDNA sequences of human, chimpanzee, gorilla, cow, mouse and rat were analyzed in rows 1, 2, 3, 4, 5, and 6 respectively. The conserved sequences across all these species are shown in row 7. The nucleotide identity is shown in different colors. Red indicates 100% identity, Blue ≥ 75, dark green ≥ 50 and light green < 50.

**Figure 4:**
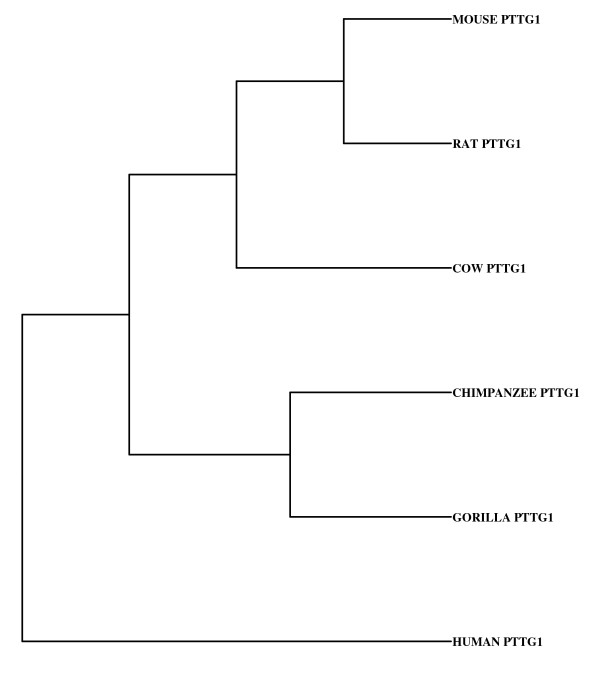
**The Neighbor phylogenetic tree analysis of PTTG from different species**. PTTG cDNA sequences of human, chimpanzee, gorilla, cow, mouse and rat were analyzed in rows 1, 2, 3, 4, 5, and 6 respectively.

The cDNA sequences of PTTG from many species are now available in the National Center of Biotechnology Information (NCBI) database. The cluster analysis of the PTTG1 cDNA sequences from human, cow, gorilla, chimpanzee, rat, and cow showed that there is 61.5% identity (consensus) in all of these species (Fig. 3). From the Neighbor phylogenetic tree analysis, it was clear that the PTTG1 cDNA from these species were clustered into two major groups that were further divided into sub and sub-sub groups (Fig. 4). The first major group consists only of human PTTG1, leaving the other species in a second group. The second group is further divided into two sub-groups in which gorilla and chimpanzee were together, which left cow, rat, and mouse in the other group. In the first sub-group, the gorilla and chimpanzee PTTG1 cDNA sequences showed 98.85% identity. The PTTG1 cDNA sequence of human and cow showed 88.34% identity, the human, chimpanzee, and gorilla sequences showed 99% identity, the human and rat sequences showed 78.65% identity, and the human and mouse sequences showed 73.73% identity. The rat and mouse PTTG1 cDNA sequences showed 81.67% identity and formed a cluster together in the phylogenetic tree.

### B. Cellular distribution

Although the role of PTTG1 as a transcriptional activator for different genes and as an inhibitor of separase makes its nuclear localization possible, a considerable amount of PTTG1 protein is localized in cytoplasm, which is still unclear. Although the hPTTG1 localizes both to the cytoplasm and to the nucleus [9,11,23,25-27], the ratio of cytoplasmic-versus nuclear localization remains controversial. Dominguez et al. [9] showed that human PTTG1 is mainly present in the cytoplasm (85%) in Jurkat cells by subcellular fractionation. Zhang et al. [11] and Seaz et al. [25] showed the predominant expression of PTTG1 in cytoplasm by *in situ *hybridization and immunohistochemistry, respectively. Stratford et al. [27] reported the predominant localization of PTTG1 in cytoplasm in HCT116 cells transfected with enhanced green fluorescent protein (EGFP)-tagged PTTG1. On the other hand, Yu et al. [26] demonstrated predominant nuclear localization of PTTG1 during interphase in JEG-3 cells when transfected with wild type PTTG1, a FLAG epitope-tagged PTTG1, or a PTTG1-EGFP construct. Interestingly, they also reported the localization of PTTG1 in some cells (<5%) to the plasma membrane. These investigators also reported the nuclear localization of PTTG1-EGFP in other cell lines like NIH3T3, rat GH3, mouse AtT20 pituitary tumor, SKOV-3 human OCA, and COS-7 monkey kidney cells. From their studies, these investigators reported the co-localization of PTTG1-EGFP at different stages of mitosis by live imaging: co-localization with microtubule asters in prophase and prometaphase in the form of granules during anaphase, which finally diminished in telophase. Mu et al. [28] showed that the sub-cellular distribution of PTTG1 is cell type-dependent. These investigators showed nuclear localization of PTTG1 predominantly in HeLa, Cos-7, and DU145 cells, but diffuse nuclear and cytoplasmic localization in A549, DLD-1, and NIH3T3 cells.

Chien and Pei [23] showed that PTTG1 was associated with PTTG1-binding factor (PBF). This association was reported to be important for PTTG1 for its translocation to the nucleus. These investigators also reported that the PBF shares significant sequence homology with a previously isolated cDNA, C21orf3 [29]. In their studies, Chien and Pei [23] also reported that PTTG1 does not contain a consensus nuclear localization signal (NLS) sequence, so it is predominantly localized in cytoplasm by indirect immunofluorescence and subcellular fractionation studies. Furthermore, they showed that the co-expression of both PBF and PTTG1 results in translocation of PTTG1 from the cytoplasm to the nucleus, suggesting that the nuclear translocation of PTTG1 requires the presence of the NLS of PBF. In the same study, the co-expression of a PBF mutant that lacks its NLS was unable to bind to PTTG1 and failed to promote PTTG nuclear accumulation.

### PTTG in cell division

#### A. Cell cycle

Equal chromosome segregation during mitosis is maintained by the separation of sister chromatids in a controlled manner. The mechanism by which chromosomes dissociate at anaphase has been solved elegantly both in yeast and mammalian cells by Uhlmann et al. [30] and Waizenegger et al. [31], respectively. The securin plays an important role in maintaining sister chromatids together until the onset of anaphase. The two sister chromatids are held together by a multisubunit cohesion complex [32]. The Smc1p, Smc3p, and sister chromatid cohesion (Scc)1p are members of the SMC family of putative ATPase proteins that are associated with chromosomes to exert a cohesive force that opposes microtubule-induced chromosome splitting [32]. Scc1p binds to chromosomes during S phase and dissociates at the onset of anaphase by a protein called separin. The premature activation of separin is prevented by the binding of securin, which is activated by the degradation of securin by anaphase-promoting complex (APC) during anaphase [33]. The APC, also called cyclosome [34], is an ubiquitin ligase (E3) complex consisting of different subunits that ubiquitinate mitotic cyclins [34], securin [10,35-37], and other cell cycle proteins [38,39]. The APC/C is activated by WD repeat proteins in a cell cycle-specific manner and the activation pattern of the APC/C is remarkably conserved from yeast to human. The APC/C is activated at metaphase and persists until the G_1 _to S-phase transition [40,41]. The APC/C is activated initially by fizzy (fzy), a *Drosophila *homologue of p55^CDC ^in rat and human, during the metaphase transition. Fzy is degraded later in mitosis (G_1 _and G_0_) and is replaced by the fzy-related (fzr) proteins that activate APC/C [42]. The fzy-mediated APC/C activity is required for the degradation of securin during the onset of anaphase, while fzr-mediated APC/C activity is essential for the degradation of mitotic cyclins, fzy, and other substrates [38,39].

#### B. Chromosomal stability

Securin protein blocks chromosome segregation in both budding and fission yeasts and in animal cells [10,35-37] and is the key substrate of the APC pathway. Paradoxically, there is also evidence that securin has a positive role in promoting sister chromatid separation. Funabiki et al. [43] showed that in fission yeast, the loss of securin completely blocked chromosome segregation and the completion of mitosis and is therefore lethal. Stratmann and Lehner [44] observed similar results in *Drosophila pimples *mutants and *pds1 *mutants in *S. cerevisiae*, which showed retarded anaphase entry [45].

The sister chromatid separation pathway, a downstream target of the mitotic spindle checkpoint, is critically important for preventing aneuploidy in the cells, which in turn can lead to cancer [46,47]. To define the role of securin in chromosomal stability, Jallepalli et al. [48] inactivated both copies of the securin gene in the HCT116 human colorectal cancer cell line by using homologous recombination. In their studies, they showed that securin is required for chromosomal stability in humans, as knockout cells exhibited a high rate of chromosome loss similar to those observed in naturally occurring cancers. Even after prolonged incubation in nocodazole or colcemid, no evidence for chromatid separation in *securin*(-/-) cells was observed. In addition, they showed that the deletion of securin blocked anaphase. This was similar to the observations reported by Funabiki et al. [43] and Stratmann and Lehner [44] in budding yeast and *Drosophila*, respectively. The time lapse experiments and immunofluorescence microscopy experiments showed that the human cells lacking securin failed to have chromatid separation. This resulted in an abnormal anaphase completion thereby creating cells with budded nuclei, chromosomal instability, and aneuploidy.

Wang et al. [49] reported that the securin in budding yeast is phosphorylated by Chk1 kinase, which may increase its stability and thereby block the cell cycle progression. Similar to Chk1, in mammalian cells, Ku-70, an enzyme involved in DNA double-strand break repair, phosphorylates PTTG *in vitro*. The occurrence of genome damaging events such as double-strand breakage can disrupt the association of PTTG1 with Ku-70 [50]. These findings were supported by Zhou et al. [51], who showed that human cells treated with DNA-damaging drugs doxorubicin and bleomycin activated p53 and suppressed PTTG1 expressions. The DNA damage activates p53, which induces cell cycle arrest for repair of the damaged DNA. In the case of damages that are beyond repair, p53 promotes programmed cell death. The functional mechanism of p53 is mainly as a transcriptional regulator that induces or inhibits expression of its target genes. The DNA damage induced by doxorubicin and bleomycin activated p53 and thereby suppressed expression of securin. Furthermore, these investigators demonstrated that activation of p53 alone is sufficient to cause repression of securin by reducing the binding of the transcription factor NF-Y to its promoter.

### PTTG in transcription

#### A. Binding elements

The expression of PTTG1 in normal tissues is restricted and found to be highly expressed in the testis in a stage-specific manner during the spermatogenesis. These studies suggest that PTTG1 may play a role in male germ cell differentiation [8,52]. Moreover, the expression levels of PTTG1 increased during cell proliferation and in mitosis in a cell cycle-dependent manner, which indicates its role in regulation of the cell cycle [53]. Although the expression levels of PTTG1 are restricted in normal cells, elevated expressions of PTTG1 were observed in many tumors including carcinomas of the lung, breast, colon, and ovary, leukemia and lymphoma, and also in pituitary adenomas [6,9,11,13,15,18,25]. Overexpression of PTTG1 in cancer cells supports the argument that PTTG1 is actively involved in cell proliferation as the cancer cell has the highest cell proliferation capacity. It is recognized that the C-terminal region of PTTG1 possesses transcriptional activity [9]. In their experiment with COS-7 cells, Chien and Pei [23] showed that when these cells were transiently transfected with fibroblast growth factor-2 (bFGF) promoter driven by luciferase along with the PTTG1 expression plasmid, the transactivation of the luciferase gene was observed (nearly 1.5-fold). On transfection of these cells, their binding partner PBF, and the PTTG1 expression vector, the transactivation of the luciferase gene was increased by 3-fold. The expression of PBF alone did not increase the reporter activity. Even though these experiments did not show direct evidence that the PTTG1 protein binds to the bFGF promoter, the reporter gene assay showed that there is a direct or indirect role of PTTG1 protein and PBF on bFGF transcriptional regulation.

Pei [54] identified the downstream targets of the PTTG1 protein. The total RNA of HeLaS3 cells expressing PTTG1 were hybridized with human cDNA expression array filters having 84 known transcripts. It was found that five gene transcripts (c-myc oncogene, MEK1, MEK3, protein kinase Cβ-1, PKCβ-1) and the heat shock protein (HSP) 70 were elevated by PTTG1 overexpression. Due to the oncogenic function of c-myc, Pei also examined the increased cell proliferation and colony formation by the induction of c-myc expression by PTTG1. Dominguez et al. [9] showed that the C-terminal portion of PTTG1 contains a transcriptional activation domain, but the direct evidence of its transactivation function by DNA-binding studies was not shown. In her experiments, Pei [54] used a c-myc promoter to characterize the PTTG1 interaction with DNA. She showed that PTTG1 protein binds to the c-myc promoter near the transcription start site and forms a complex with the ubiquitous transcription activator upstream stimulating factor 1 (USF1). Mapping of PTTG1 protein showed the region between amino acids 60 and 118 as the PTTG-DNA binding domain. Later on, Stratford et al. [27] identified PBF expression in thyroid tumors and demonstrated that PBF had a transforming ability *in vitro *and a tumorigenic ability *in vivo*. The PTTG1 and PBF expressions were upregulated in thyroid cancer [27,55,56] and were reported to stimulate bFGF expression [11]. Taking this into account as well as the PTTG1 role in repressing iodide uptake *in vivo *[56], Boelaert et al. [55] studied the role of PTTG1 and PBF in the repression of sodium iodide symporter (NIS) expression and function in thyroid tumors. From their *in vitro *experiments, it was observed that the PTTG1 and PBF inhibit NIS mRNA expression and iodide uptake. In their detailed studies on the NIS promoter in rat FRTL-5 cells, they transfected pGL3-luc promoter constructs, which contain either 544 base pairs (bps) of the proximal NIS promoter, the human NIS upstream enhancer element (hNUE), or a fusion of both (hNUE-basal NIS) co-transfected with PTTG1 or PBF. From these experiments, they observed no significant reduction in reporter activity when transfected with NIS basal promoter with PTTG1, but did observe a significant reduction in reporter expressions when transfected with PTTG1 and hNUE as well as its fusion with NIS. In contrast, PBF could significantly suppress the promoter activity with the NIS basal promoter, hNUE, and also the fusion promoter of both NIS-hNUE. They also showed that within this ≈1 kb element, the USF1 response element is critical to PTTG1 whereas PBF requires the USF1/PAX8 complex for repression of NIS.

#### B. Interacting proteins and the pathways

To understand the role of human PTTG1 in sister chromatid separation and tumorigenesis, Romero et al. [50] utilized a yeast two-hybrid approach to identify the proteins that interact with PTTG1. In their experiment, they isolated a protein of 70 kDa, Ku-70, which specifically interacts with PTTG1. The Ku-70 protein interacts with Ku-80, which together forms the DNA-dependent protein kinase (DNA-PK) [57]. This enzyme is involved in DNA double-strand breakage repair caused by certain chemical and genetic reactions including some chemotherapeutic drugs. Romero et al. [50] showed that the Ku dimer interacts with the N-terminal portion of human PTTG1. In their experiments, they also demonstrated that DNA double-strand breakage prevents PTTG-Ku-70 interaction by activation of DNA-PK complexes. The DNA-PK complex phosphorylates PTTG1, which blocks the sister chromatid separation. These findings support the role of human PTTG1 in tumorigenesis by causing aneuploidy through DNA damage-response pathways.

High levels of PTTG1 expression have been reported in a variety of tumors. It is also known that insulin-like growth factor-I (IGF-I) and insulin induce many oncogenes [58]. Chamaon et al. [17] tested the influence of IGF-I and insulin on PTTG1 expression in human astrocytoma cells in comparison to proliferating non-neoplastic rat embryonal astrocytes. PTTG1 mRNA expression and protein levels were increased in malignant astrocytes when treated with IGF-I or insulin, whereas in rat embryonic astrocytes, PTTG1 expression and protein levels increased only when cells were exposed to IGF-I. In their experiments, they showed that the IGF-I/insulin-pathways regulate PTTG1 transcription. In our experiments using the breast tumor cell line MCF-7, we showed that insulin and IGF-1 regulate the expression of PTTG1 primarily through the activation of phosphoinositol-3-kinase (PI3K)/AKT cascade [59]. Heaney et al. [60] showed a 2.4-fold induction of PTTG1 mRNA in NIH-3T3 cells when treated with bFGF. In addition to these findings, Tfelt-Hansen et al. [61] showed that the stimulation of the U87MG cell line with EGF and TGFα upregulated PTTG1 expression. Voltides et al. [62] showed that PTTG1 is a target for EGFR-mediated paracrine regulation of pituitary cell growth. We also showed the suppression of bFGF in H1299 tumors when treated with PTTG1 small interfering (si)RNA compared to untreated and control-treated siRNA in nude mice [18]. Taken together, it was suggested that PTTG1 regulates and/or is regulated by many different growth factors such as IGF-I, EGF, TGFα, and bFGF, which are involved in pathways such as PI3K, mitogen-activated protein kinase (MAPK), and angiogenesis.

During our recent investigations to understand the mechanisms by which PTTG1 is involved in tumor angiogenesis and metastasis, we performed transient and stable transfections of HEK293 cells with PTTG1 cDNA and studied the expression and secretion of matrix metalloproteinase (MMP)-2 [18]. The zymography, reverse transcriptase polymerase chain reaction (RT/PCR), ELISA, and MMP-2 gene promoter activity assays showed significantly increased MMP-2 secretion and expression. We also showed a significant increase in cell migration, invasion, and tubule formation of human umbilical vein endothelial cells (HUVECs) when treated with the conditioned medium collected from the HEK293 cells overexpressing PTTG1. Based on these experiments, we suggest that PTTG1 is actively involved in tumor angiogenesis and metastasis via activation of proteolysis and increases in invasion occur through modulation of MMP-2 activity and its expression. Blocking or down regulation of PTTG1 in tumors may result in suppression of tumor growth and metastasis through the related down regulation of MMP-2 expression and activity.

### PTTG in cancer

#### A. Tumor initiation and cell proliferation

**Figure 5:**
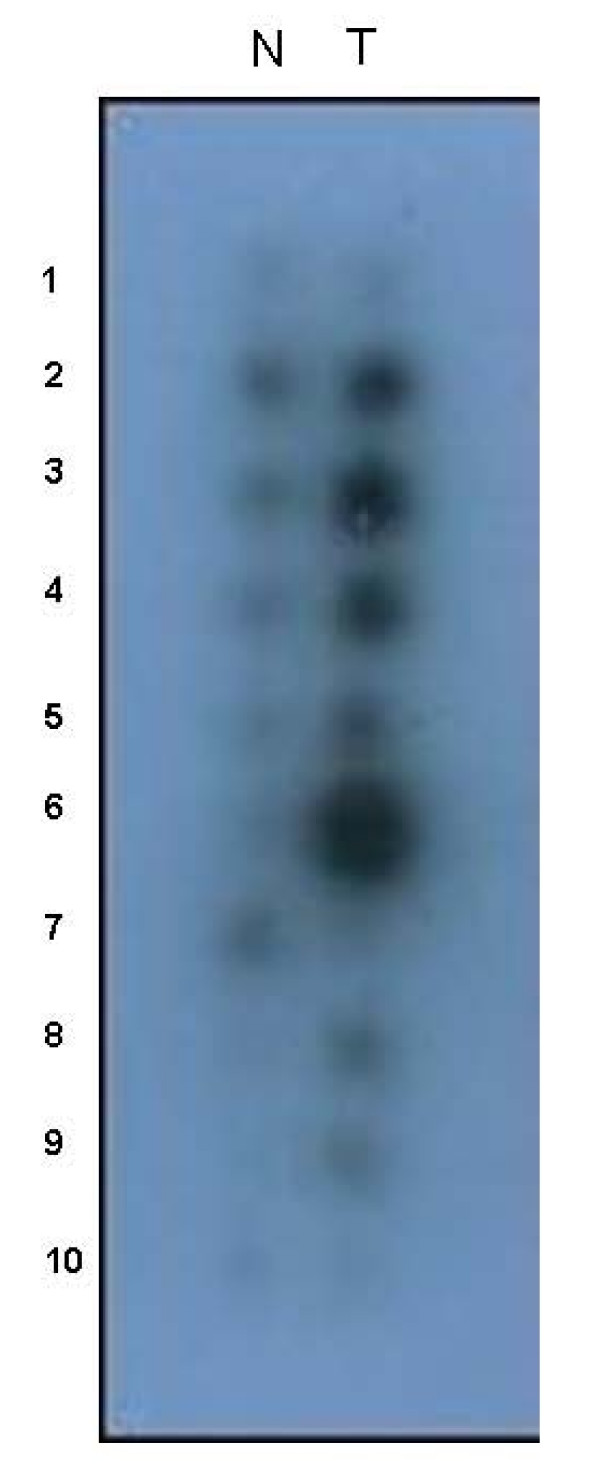
**The northern blotting analysis of PTTG expression in various ovarian cancer patients**. N indicates normal ovarian tissue and T indicates the ovarian cancer tissue from the corresponding patient. The numeric 1, 2, 3, and so on indicates the tissues different patients.

Since PTTG1 was isolated and characterized, there have been nearly 100 articles published on the role of PTTG1 in various cancers. Many investigators have focused on determining the mechanisms and pathways by which PTTG1 induces its tumorigenic function. Pei and Melmed [7] showed that overexpression of PTTG1 in mouse NIH3T3 fibroblasts inhibited cell proliferation and induced cell transformation *in vitro*. Injection of PTTG1 transfected NIH3T3 cells into athymic nude mice resulted in tumor formation within 3 weeks in all these animals. From their results, they suggested that this gene might play a role in pituitary tumorigenesis. Dominguez et al. [9] cloned a human cDNA homologue of PTTG1 (hpttg). They reported that the expression of hpttg in samples obtained from normal donors was very low or undetectable, whereas it was found to be overexpressed in Jurkat cells as well as in leukocytes from patients with different kinds of hematopoietic neoplasms or myelodysplastic syndromes. Zhang et al. [11] investigated the expression levels of PTTG1 in many normal and cancerous cells and found that PTTG1 is expressed in normal adult testis, thymus, colon, small intestine, brain, lung, and fetal liver. We also observed the overexpression of PTTG1 in tissues of ovarian cancer from different patients (Figure. 5). It is expressed most abundantly in several carcinoma cell lines including cervix carcinoma HeLa cell, choriocarcinomas JEG-3 and JAR, breast adenocarcinoma MCF-7, osteogenic sarcoma U-2OS, hepatocellular carcinoma Hep 3B, lung carcinoma EY, ovarian CAOV3 and thyroid carcinoma TC-1. Saez et al. [25] also isolated and characterized hpttg from human thymus and studied the expression of hpttg in human pituitary adenomas. In their studies, they found that the hpttg is highly expressed in the majority of pituitary adenomas while only very low levels were detected in normal pituitary glands.

Later, the overexpression of this gene was reported in many other tumors including esophageal cancer [63], thyroid cancer [55], small cell lung cancer and non-small cell lung cancer [18,64,65], testicular cancer [15,66], OCA [6,15,67], breast cancer [14,59], uterine leiomyomas [16], liver cancer [68], and colorectal cancer [69]. Kakar and Jennes [12] and Hamid et al. [70] showed that overexpression of PTTG in NIH3T3 as well as the human embryonic kidney cell line HEK 293 resulted in increased cell proliferation, induction of cellular transformation, and development of tumors in nude mice, suggesting an oncogenic function of PTTG in human tumorigenesis.

#### B. Angiogenesis

Soon after the isolation and characterization of PTTG1 in humans, Heaney et al. [60] reported that PTTG1 is regulated *in vivo *and *in vitro *by estrogen and that its induction levels coincide with bFGF and vascular endothelial growth factor (VEGF). The bFGF and other growth factors are known to modulate angiogenesis in many tissues; regulation of expression of these factors by PTTG1 suggests a role of PTTG1 in angiogenesis. Ishikawa et al. [71] showed that the conditioned medium collected from the NIH3T3 cells transfected with wild type human PTTG1 induced angiogenesis. They also observed that the bFGF concentration in PTTG1 conditioned medium was elevated compared to the conditioned medium from untransfected NIH3T3 cells. From their experiments, they concluded that the human PTTG1 induces an angiogenic phenotype via bFGF both *in vitro *and *in vivo*. Kim et al. [72] investigated the role of PTTG1 in regulating angiogenic factors in addition to VEGF and bFGF in thyroid cancer. As specified above, the PTTG1 has been shown to up-regulate VEGF expression. It has also been reported that VEGF may up-regulate an angiogenic gene known as an inhibitor of DNA binding-3 (ID3) expression [73], which is believed to play a critical role in cell proliferation and to be a precursor of endothelial cell recruitment [74,75]. As the ID3 is differentially expressed in thyroid cells, which also express high PTTG1 levels, an interaction between PTTG1, VEGF, and ID3 is suggested. Recently, Kim et al. [72] demonstrated the suppression of the angiogenic inhibitor thrombospondin-1 (TSP-1) by PTTG. They also showed regulation of expression of ID3 by PTTG in primary human thyroid cells. The mechanisms by which PTTG1 regulates expression of TSP-1 and ID3 are not clear. However, these investigators suggest that the mechanisms by which PTTG1 regulates TSP-1 and ID3 expression may be direct or indirect, but it remains unclear along which pathway PTTG1 exerts this effect. Furthermore, these investigators showed that the effects of PTTG1 on ID3 expression were significantly reduced when they used the SH3 domain mutant of PTTG1, suggesting an important role for the SH3 domain in regulating ID3 expression. The SH3-binding domain of PTTG1 has been shown to be involved in up-regulation of both VEGF and FGF-2 [76,77]. The C-terminal portion of PTTG1 contains a DNA-binding domain that is shown to be involved directly in stimulating the c-*myc *promoter [54], suggesting that a direct interaction of PTTG1 with the ID3 gene may contribute in part to PTTG1's regulatory effect.

In addition to ID3, Kim et al. [72] also showed that the angiogenic inhibitor TSP-1 is decreased by 2.5-fold in response to PTTG1 overexpression *in vitro*. Suppression of PTTG1 with siRNA a 2-fold induction of TSP-1 was observed. From these observations, these investigators concluded that PTTG1 may promote tumor angiogenesis by regulating the expression of angiogenic genes such as VEGF, bFGF, ID3, and TSP-1, suggesting that PTTG1 may be a key gene in thyroid tumorigenesis. Although the complete mechanism of PTTG1 in angiogenesis is not known, it appears that PTTG1 is an important gene in regulating several angiogenic genes by multiple pathways. Therefore, detailed studies on PTTG1 in angiogenesis are essential for developing a stage-specific as well as a targeted cancer therapy.

#### C. Metastasis

As described above, PTTG1 plays an important role in angiogenesis and there are many reports on its involvement in metastasis. Shibata et al. [63] examined PTTG1 expression levels in esophageal cancer. They observed significantly higher PTTG1 mRNA levels in tumor tissues compared to the corresponding normal tissues. They further showed a correlation between expression and levels of pain and with pathological stage and extensive lymph node metastasis. These investigators also observed the median survival time (8.5 months) for patients with high PTTG1 expression levels compared to the survival time (14.0 months) for patients with low PTTG1 expression levels, suggesting a role of PTTG1 in metastasis. Solbach et al. [14] analyzed 72 tumor samples derived from primary tumors of patients suffering from breast cancer and unaffected breast epithelium for PTTG1 mRNA expression levels and to determine a relationship with pathological parameters over a 5-year observation period. From their analyses, these investigators found a direct correlation between PTTG1 mRNA overexpression and lymph node infiltration. The overexpression of PTTG1 in tumors correlated with a higher degree of tumor recurrence and tumor aggression. They also obtained similar results with primary tumors of 89 patients suffering from squamous cell carcinoma [78]. Consistent with these observations, Ramaswamy et al. [79] reported a correlation between expression levels of PTTG1 with metastatic adenocarcinomas using microarray analysis.

To understand the mechanism of PTTG1 in angiogenesis and metastasis, Malik and Kakar [80] studied the regulation of MMPs by PTTG1. MMPs are the proteolytic enzymes required for tumor cells to invade and metastasize. MMPs are known to play a key role in degradation of the basement membrane and extracellular matrix. Among these, MMP-2 and MMP-9 cleave the type IV collagen and gelatin, which are the principal structural components of the basement membrane [81]. Since PTTG1 has been reported to have a role in angiogenesis and metastasis [19,55,79], we hypothesize that the regulation of MMP-2 by PTTG1 is one of the mechanisms by which PTTG1 contributes to angiogenesis and metastasis.

#### D. Apoptosis

PTTG1 has been shown to regulate the expression of p53, a tumor suppressor gene that subjects to apoptosis by inducing apoptosis-inducing genes. Yu et al. [26] hypothesized the role of PTTG1 in apoptosis. In their studies, they examined p53 involvement in PTTG1-induced cell death in cells expressing or lacking wild type p53. In their experiments, these investigators showed that in MCF-7 breast cancer cells expressing wild type p53, PTTG1 overexpression caused apoptosis. They observed translocation of p53 to the nuclei in cells that overexpressed PTTG1. In their experiments, they also observed that the dominant negative p53 mutant did not inhibit PTTG1-induced apoptosis in MCF-7 cells. From these observations, they concluded the mutant p53 may not completely inhibit endogenous p53 activity and that mechanisms other than p53 may also be involved in PTTG1-induced apoptosis. They also observed apoptosis by PTTG1 in p53-negative MG-63 cells. The transient or stable expressions of PTTG1 in these cells were found to increase the frequency of aneuploidy. Based on these experiments, these investigators concluded that aneuploidy was not the cause of apoptosis in these PTTG1 overexpressing cells since the majority of cells were not aneuploid before they entered apoptosis. They suggested that PTTG1 has a duel role in inducing p53-dependent and p53-independent apoptosis as well as aneuploidy. In the cells where both of the apoptotic pathways fail, PTTG1 induces these cells to undergo aneuploidy, which eventually results in cancer.

The overexpression of p21^WAF1/CIP1 ^was shown to cause both G1/S and G2/M arrest and can effectively suppress tumor growth [82,83]. The p21^WAF1/CIP1 ^is a cyclin kinase inhibitor that can induce cell growth arrest by inactivating cyclin-dependent kinase (Cdk) or by inhibiting the activity of proliferating cell nuclear antigen (PCNA). The serum and various growth factors such as PDGF, FGF, EGF, and TGF-β can induce p21^WAF1/CIP1 ^expression [84-86]. Mu et al. [28] investigated the role of PTTG1 in p21^WAF1/CIP1^-induced apoptosis in the A549 lung cancer cell line. In their studies, they overexpressed PTTG1 in HeLa and A549 cells and showed inhibition of cell proliferation, colony formation, and thymidine incorporation. They observed no change in the levels of expression of p53 and p21^WAF1/CIP1 ^in PTTG1 overexpressed HeLa cells, whereas the p21^WAF1/CIP1 ^was overexpressed by PTTG1 in A549 cells. No significant change in p53 expressions was observed in either cell line transfected with PTTG1. These investigators also studied the effect of PTTG1 on p21^WAF1/CIP1 ^expression by the luciferase assay. They showed activation of the lucifierase gene driven by p21^WAF1/CIP1 ^promoter by PTTG1 cDNA, suggesting that the overexpression of PTTG1 in A549 caused p21^WAF1/CIP1^-mediated apoptosis, which is an event independent of p53-mediated apoptosis. However, the p21^WAF1/CIP1 ^expression levels did not increase significantly in HeLa cells overexpressed with PTTG1, suggesting that PTTG1 can mediate apoptosis both by p21^WAF1/CIP1^-dependent and independent pathways.

### Mouse models

#### A. Transgenic mouse model

To understand the role of PTTG1 in tumorigenesis, Abbud et al. [87] developed transgenic mice expressing PTTG1 under the control of mouse αGSU promoter. These transgenic mice showed PTTG1 expression in LH- and GH-producing cells ranging from hyperplasia to development of frank adenoma. The expression of PTTG1 in GH cell hyperplasia was surprising because the αGSU promoter has not been shown to express in mature somatotropes. In addition, transgenic mice showed prostate and seminal vesicle neoplasia due to increased GH and LH that resulted in elevated IGF-I and testosterone levels. In males, they observed bladder obstruction, kidney reflex, and inflammation due to prostate hyperplasia. The transgenic male mice also showed multiple reproductive defects similar to transgenic mice that overexpress human chorionic gonadotropin [88,89], suggesting its role in LH overexpression leading to prostate hyperplasia. This also suggests that PTTG1 overexpression in transgenic mice affects early pituitary multipotential cells or may influence neighboring non-αGSU-expressing cells by some unknown paracrine mechanism.

The oncogenic function of PTTG1 was further confirmed by crossbreeding of retinoblastoma (*Rb*; +/-) animals with transgenic animals [90]. *Rb *is an important tumor suppressor gene, one that is phosphorylated by G1 cyclin/Cdk complexes. The mice bearing a single *Rb *mutant allele developed pituitary tumors with almost complete penetrance [91-93]. Chesnokova et al. [90] observed high levels of pituitary PTTG1 mRNA and protein in pretumorous *Rb*^- ^mice. Crossbreeding of *Rb *(+/-) animals with PTTG1 transgenic animals showed enlarged pituitary glands and a 3.5-fold increase in the frequency of tumors originating fromα-subunit-expressing cells. They also observed that bitransgenic animals with enlarged pituitary glands developed pituitary tumors earlier compared to *Rb *(+/-) animals. Confocal microscopic experiments revealed an alteration in the chromatin pattern similar to malignant cells. Increases in pituitary hyperplasia in PTTG1 overexpressing cells were observed in these bitransgenic mice when compared to *Rb*+/- mice, supporting a role of PTTG1 in pituitary gland tumorigenesis. In their studies, these investigators found an absence of the antiapoptotic marker bcl-2 in these animals, suggesting that there might be a different mechanism other than inhibition of the apoptotic pathway.

Almost 90% of OCAs are derived from the ovarian surface epithelium (OSE). However, the biology of epithelial cancer initiation remains unknown. As reported above, PTTG1 is found to be overexpressed in most of the ovarian tumors analyzed to date and the PTTG1 promoter is specifically activated in tumor cells, including OSE tumor cells. To evaluate the oncogenic function of PTTG1 and its ability to transform OSE cells, we generated transgenic animals that express PTTG1 in OSE cells under the control of the MISIIR gene promoter [6]. We generated an F1 population by breeding the male F0 founders with wild type females. Analysis of the male transgenic animals revealed urinary tract obstruction secondary to prostate hyperplasia (enlarged prostate) at 8 to 10 months of age. The urinary bladder was enlarged with wall thickening, increased in visualization, and filled with urine containing white deposits [6]. The seminal vesicles and epididymis in some of the transgenic males were enlarged. Preliminary observation of the seminal vesicles showed signs of hyperplasia or adenoma.

**Figure 6:**
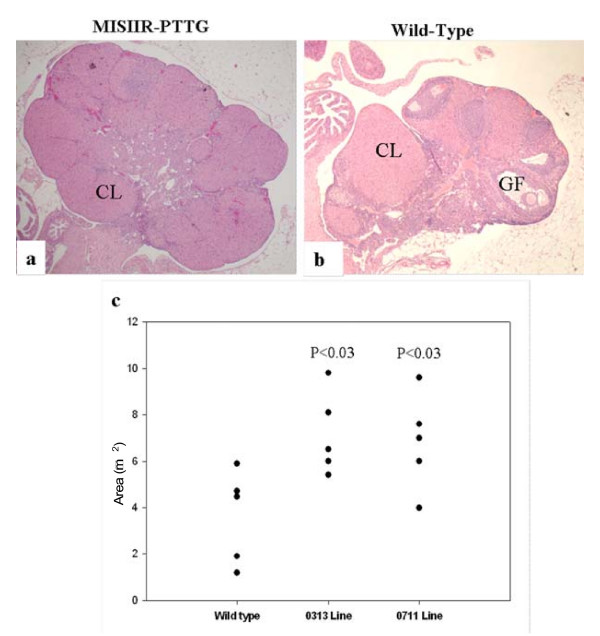
**Histopathological analysis of the ovaries from transgenic and non-transgenic animals**. Transgenic animals showed enlarged ovaries, and presence of large number of corpus lutea compared to non-transgenic animals. The size of the ovaries in μm^2 ^from transgenic and non-transgenic animals was determined by measuring the length and width of histological sections at 4× magnification using Metmorph Software version 6.2. The figure was reproduced with permission from J Endocrinology (6).

**Figure 7:**
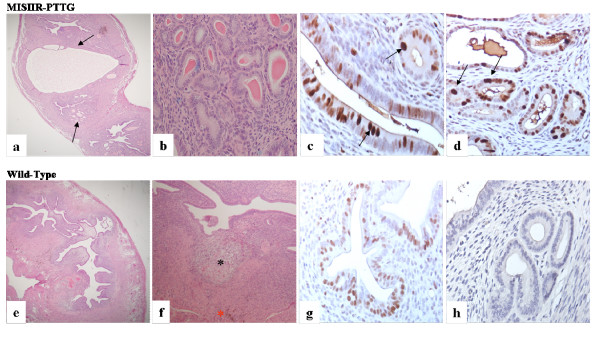
**Histological analysis of PTTG transgenic uterus**. The uteri from transgenic mice (a and b) had large cysts and multiple small fluid-filled glandular cysts compared to non transgenic mice (e and f). Scar formation and hemosiderin laden granules (asterisk in f) were evident in wild-type but absent in transgenic uterus. Both wild-type and transgenic uteri were PCNA positive; PCNA staining in transgenic uteri of transgenic animals was much darker and homogeneous compared to non-transgenic animals, suggesting the presence of highly proliferative cells both in luminal and glandular epithelium. Negative control sections in which primary antibody were omitted did not display any PCNA staining (h). The figure was reproduced with permission from J Endocrinology [6].

Analysis of female transgenic mice showed expression of transgene in OSE cells. The transgenic females (8 to 10 months old) had enlarged and abnormal ovaries (Fig. 6). Instead of developing oocytes in various cycles, they all seem to be at the corpora luteal stage. In addition, the ovaries appeared to be enlarged with an increased number of corpora luteum. Only rare primary follicles were noted compared to age-matched, non-transgenic mice, in which the ovaries showed follicles at various stages of development. The fallopian tube glandular structure demonstrated less complexicity compared to normal and the fallopian tubes of the transgenic mice no longer showed vasculation (Fig. 7). The endometrium of transgenic mice showed various abnormalities including cystic dilation of the uterine cavity as well as increased complexicity of the endometrial glands with increased secretion as compared to non-transgenic mice. There appeared to be no evidence of a previous cycle, such as scaring, hemosiderin-laden macrophages, or stromal histocytes. The increase in the number of corpora luteum in transgenic mice could be due to increases in LH leading to increases in ovulation.

**Figure 8:**
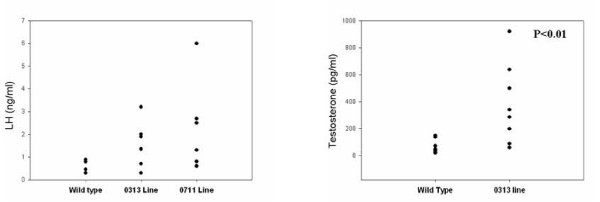
**LH and testosterone levels in transgenic and wild-type animals**. Serum concentration of LH (A) and testosterone (B) were measured in 8- to 10-month old transgenic and wild-type females using EIA kit. The data was reproduced with permission from J Endocrinology [6].

Measurement of serum LH and testosterone using the EIA kit showed a significant change in the levels of LH and testosterone (Fig. 8) in two lines compared to wild-type animals, whereas no change in FSH levels were observed. Estrogen levels showed a 1.3-fold increase, but progesterone levels remained unchanged (data not shown). Although there appeared to be early signs of hyperplasia and neoplasia, however, none of the animals developed visible ovarian or endometrial tumors within 8- to 10-months of age, suggesting that PTTG1 overexpression is not sufficient to induce tumorigenesis in mice and requires a hormonal trigger or cooperation of other gene(s), such as the inactivation of p53, to initiate tumorigenesis in OSE cells.

#### B. Knockout mice

To further demonstrate the importance of PTTG1 in tumorigenesis and other biological functions, Wang et al. [94] developed mice lacking the murine PTTG1 gene by homologous recombination. They observed PTTG1 -/- mice for up to 8 months and found that these animals were viable and fertile in contrast to the phenotypes observed in yeast or *Drosophila *securin. As securin is involved in inhibition of sister chromatid separation, it was expected that PTTG1-null mice may not survive. However, the non-fatal phenotype of these knock out mice suggests that there might be more than one mechanism for sister chromatid separation. Thus, it is likely that the other mechanisms compensated for the loss of securin and its function in the cell cycle. The mice lacking PTTG1 were found to have testicular and splenic hypoplasia, thymic hyperplasia, and thrombocytopenia. From these results, the investigators suggested that the PTTG1 disruption caused cell cycle and chromosomal changes in PTTG1-abundant tissues in a tissue-specific manner and that PTTG1 is involved in spermatogenesis and platelet formation. In their subsequent studies, Wang et al. [95] showed that the reduction of pancreatic islet mass and the decrease in β cell numbers in PTTG1 knockout male mice resulted in diabetes type I in their late adulthood. Though the IGF-1 and thyroid hormone levels were normal in these mice, some additional non-genetic factors may have contributed to hyperglycemia development in these mice in addition to PTTG loss. In contrast, PTTG -/- female mice exhibited normal plasma glucose levels, suggesting that estrogen might be protective for islet maintenance and in β cell proliferation. To define the role of PTTG1 in tumorogenesis, Chesnokova et al. [90] crossbred PTTG1-null transgenic mice with *Rb *(+/-) mice. They showed that by crossbreeding *Rb *(+/-) mice with knockout mice, the development of pituitary tumors could be delayed. In their experiments, they suggested that the absence of PTTG1 may allow expression of p21, which causes cell cycle arrest. These results support the findings of Abbud et al. [87], who showed that overexpression of pituitary PTTG1 in *Rb *(+/-) animals influenced tumor initiation and progression by enhancing cell proliferation.

### A target for cancer therapy

#### A. PTTG siRNA

As described above, PTTG1 has an important role in the cell cycle and tumorigenesis through different pathways. In this section, we describe the possibilities of exploring PTTG1 for targeted cancer therapy. Various researchers have found an abnormally high expression of PTTG1 in a wide range of human primary tumors such as those found in the ovary, testis, kidney, colon, thyroid, pituitary, liver, adrenal, and breast as well as in a range of tumor cell lines such as melanoma, leukemia, and lymphoma [9,12,15,19,25,53,55,63,79,96-98]. These findings indicate that PTTG1 may be involved in tumorigenesis in humans. Further supporting this idea, high expression levels of PTTG1 have a positive correlation with increased tumor invasiveness in human pituitary tumors [98] and degree of malignancy, pathogenesis, and progression of colorectal, thyroid, and breast tumors [19,55,97]. PTTG1 has been identified as one of eight signature genes upregulated in human primary tumors that are associated with tumor metastasis [79].

Much has been investigated and is known about the upregulation of PTTG1, downregulation of PTTG of keen interest at this point. Kakar and Malik [18] used interference RNA (RNAi) directed against PTTG1 to study this effect. In this technique, siRNA is introduced into cells to silence the expression of a gene. The siRNA is a 19-nucleotide long, double-stranded RNA that enters the cell and degrades the mRNA of the target gene [99]. We [18] transfected H1299 cells with PTTG1 siRNA duplex and found that PTTG1 mRNA was almost completely depleted within a day or two in comparison to untransfected or control cells. We also found that there was almost a 60% reduction in PTTG1 protein after two days of transfection. When H1299 cells transfected with PTTG1 siRNA duplex were plated on soft agar, colony formation was significantly reduced compared to untransfected or control cells. Mice injected with H1299 cells transfected with PTTG1 siRNA followed by intratumoral injection of PTTG1 siRNA, mice developed no tumors after two weeks and small tumors after four weeks compared to large tumors that developed in mice injected with untransfected or control siRNA transfected cells. PTTG1 siRNA was successful in downregulating PTTG1 as well as Ki67, bFGF, and CD34 in H1299 tumors in nude mice. These results indicate that downregulation of PTTG1 using PTTG1 siRNA inhibits tumors grown *in vitro *and *in vivo*.

In another study, El-Naggar et al. [100] used specific siRNA to down regulate PTTG1 expression. These investigators silenced PTTG1 expression in human A2780 OCA cells and investigated the effect on tumor formation *in vitro *and *in vivo*. The mRNA and protein of PTTG1 expression was significantly reduced by treatment with siRNA. Stable cell lines expressing PTTG1 siRNA showed a 50% reduction in cell proliferation compared to vector or control transfected cells and colony formation was reduced by 70% in soft agar. Nude mice injected with A2780 cells expressing PTTG1-siRNA showed a decrease in tumor development and growth [100].

#### B. PTTG Adenovirus siRNA

Adenoviruses were originally identified and characterized as pathogens that cause common colds, gastroenteritis, conjunctivitis, cystitis, or respiratory illnesses. Later, these adenoviruses were explored in the investigation of mammalian molecular and cell biology experiments as well as for becoming an efficient vector for gene therapy [101]. Although there are many other viral vectors such as retroviruses, adeno-associated viruses (AAVs), lentiviruses (LVs), and herpes simplex viruses (HSVs) used for cancer and other gene therapies, the high infectivity of most mammalian cell types, high expression level of the transgene, ease for achieving high virus titer and replication deficiency, large capacity for accommodating transgene(s), and non-integration into the mammalian genome have made adenovirus one of the most widely used gene transfer vectors [102]. The replication-defective adenovirus has been commonly used as a vector for cancer gene therapy to transfer tumor-suppressor genes, anti-angiogenic factors, prodrug-activating genes, and immunostimulatory genes [102-104]. Recently, Cho-Rok et al. [68] utilized the adenovirus vector system to deliver PTTG1 siRNA and showed the inhibition of liver cancer cell growth both *in vitro *and *in vivo*. In their experiment, they showed that PTTG1 serves as a negative regulator of p53 tumor suppression in hepatocellular carcinoma (HCC) and that depletion of PTTG1 significantly inhibits growth of PTTG1-overexpressing HCC cell lines *in vitro *and *in vivo*. From these experiments, it is evident that the adenovirus-mediated PTTG1-siRNA appears to selectively target tumor cells expressing high levels of PTTG1 with functional p53, which may serve as a valuable gene therapy approach for treating human cancers including ovarian cancer. Taken together, these findings strongly suggest that down regulation of PTTG1 in cancer results in suppression of tumor growth and progression. Therefore, down-regulation of PTTG1 or inactivation of its tumorgenic function in cancer may become an important target in developing new cancer therapies.

## Conclusion

**Figure 9:**
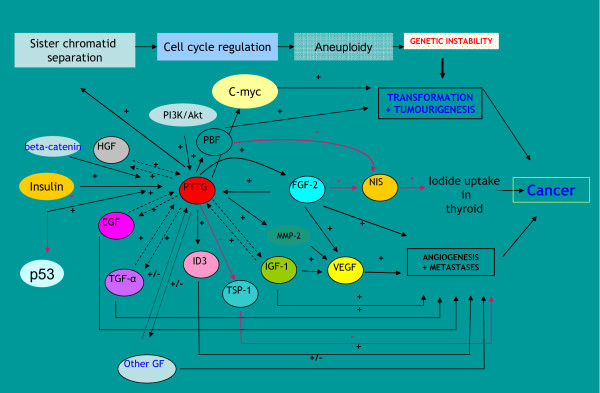
**Regulation of PTTG expression by various growth factors and predicted biological and tumorigenic functions**. The regulatory factors and pathways regulated by PTTG are shown.

PTTG is an oncogene with multiple domains and multiple functions. It is transcriptionally regulated by various growth factors and is highly expressed in most of the tumors and tumor cell lines analyzed to date. Up regulation of PTTG increases cell proliferation, induces cellular transformation and promotes tumor development in nude mice. On the other hand down regulation of PTTG in cancer results in suppression of tumor growth and angiogenesis, suggesting that PTTG may serve as an important target gene for the treatment of cancer. The molecular mechanism by which PTTG mediates its tumorigenic function is still unclear. The possible mechanism and pathways by which PTTG induces its tumorigenic functions are summarized in Fig. 9.

## Competing interests

The authors declare that they have no competing interests.

## Authors' contributions

SKP and CY drafted the manuscript. SSK participated editing of the manuscript. All authors read and approved the final manuscript.
